# Experimental data on the air-sea energy fluxes at the tropical coastal ocean in the southern South China Sea

**DOI:** 10.1016/j.dib.2018.06.020

**Published:** 2018-06-19

**Authors:** Yusri Yusup, John Stephen Kayode, Abbas F.M. Alkarkhi

**Affiliations:** a Environmental Technology, School of Industrial Technology, Universiti Sains Malaysia, USM 11800, Pulau Pinang, Malaysia; b Centre for Marine & Coastal Studies(CEMACS), Universiti Sains Malaysia, Pulau Pinang, Malaysia; c Malaysian Institute of Chemical & Bioengineering Technology, Universiti Kuala Lumpur, 78000 Melaka, Malaysia

## Abstract

Air-sea flux exchanges influence the climate condition and the global carbon-moisture cycle. It is imperative to understand the fundamentals of the natural systems at the tropical coastal ocean and how the transformation takes place over the time. Hence, latent and sensible heat fluxes, microclimate variables, and surface water temperature data were collected using eddy covariance instruments mounted on a platform at a tropical coastal ocean station from November 2015 to October 2017. The research data is to gain the needful knowledge of the energy exchanges in the tropical climatic environment to further improve predictive algorithms or models. Therefore, it is intended that this data report will offer appropriate information for the Monsoonal, and diurnal patterns of latent (LE) and sensible (H) heats and hence, establish the relationship between microclimate variables on the energy fluxes at the peninsular Malaysian tropical coastal ocean.

**Specifications Table**

**Table:** 

Subject area	*Meteorology and Environmental Science*
More specific subject area	*Environmental and Climate Change*
Type of data	*Excel file and figures*
How data was acquired	*Half-hourly measurements latent and sensible heat fluxes and other microclimate variables using; (1) infra-red carbon dioxide and water vapor analyzer (LI-7550); (2) 3D sonic anemometer (RM81000); (3) net radiometer (NR LITE 2); (4) pyranometer (LI-200SL); (5) temperature and humidity sensor (HMP155), and (6) thermistor (LI-COR).*
Data format	*Filtered and quality-controlled*
Experimental factors	*Half-hourly measurements in the air-sea interface*
Experimental features	*Data post-processing was achieved using a computer program, EddyPro® (Version 6.2.0, LI-COR, Inc., USA) to process the data as 20 Hz time series.*
Data source location	*Straits of Malacca, South China Sea; Pulau Pinang, Malaysia at latitude 5°28’06’’N, and longitude 100°12’01’’E*
Data accessibility	*The data is available with this article as a supplementary Excel file.*

**Value of the data**

• This data showed that the tropical coastal ocean energy exchanges between the atmosphere and the ocean biospheres is dynamic in nature and thus not easily predicted by the applications of the existing TOGA-COARE models.
• Data of this nature are very important for researchers working on the relationship between microclimate variables and the energy budgets.
• The research data is related to the government policy of improving the environmental and health conditions of the coastal and estuaries in any part of the world.

## 1 Data

**Fig. 1:**
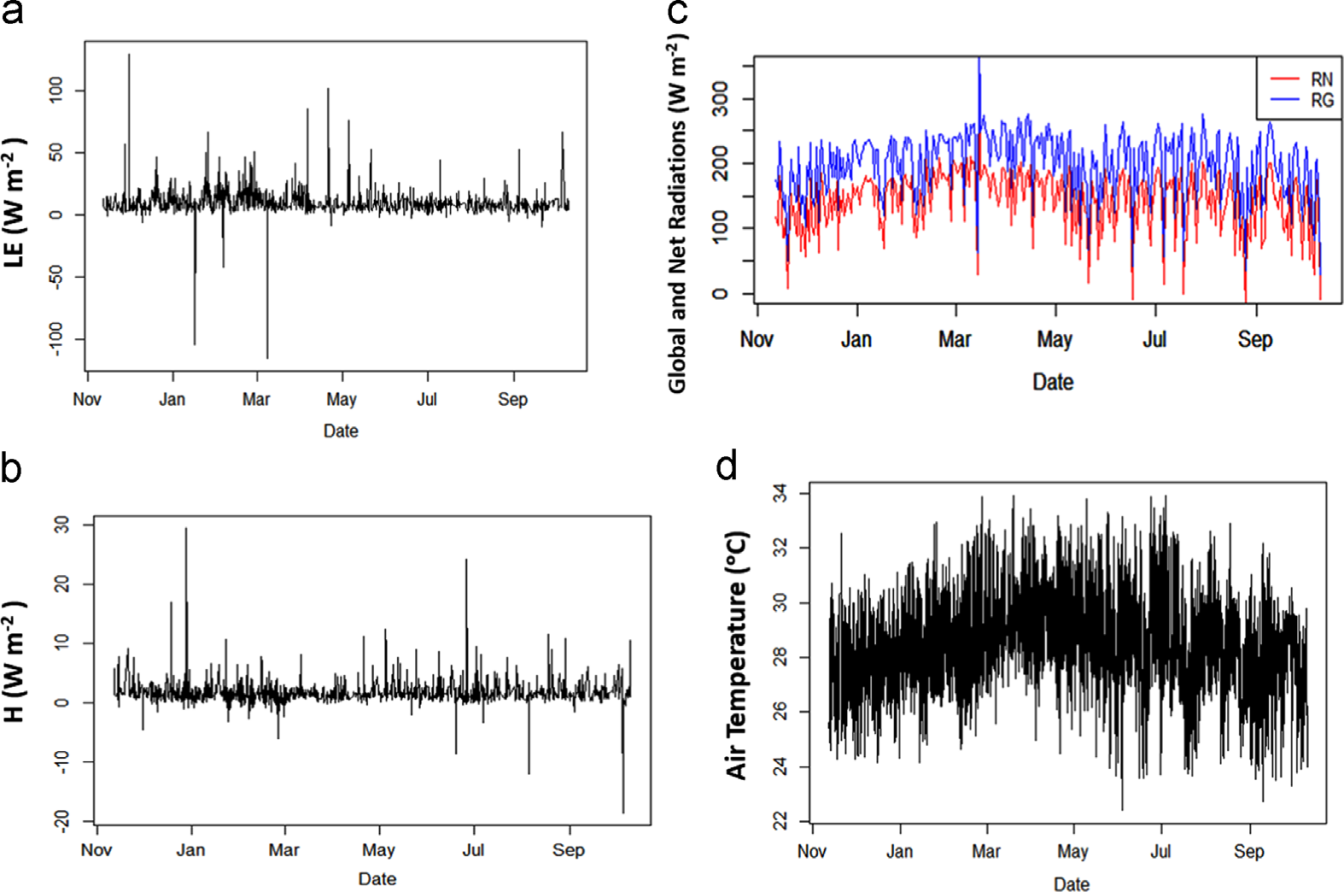
The raw data in W m^−2^, of (a) latent heat flux, (LE) and (b) sensible heat flux, (H) (c) global (R_G_) and net (R_N_) radiations, and (d) the Air temperature in °C recorded at the tropical coastal station (5°28′6′′N, 100°12′1′′E).

The tropical coastal ocean plays a significant role in the energy exchange between the air and sea compared to higher and lower latitude regions due to increased and persistent solar radiation and high and constant water surface temperature [1]. The scarcity of high-quality flux data in the tropical region, such as in the southern South China Sea, results in the deficiencies of understanding of the air-sea interaction in the tropical coastal ocean. Locations of highest exchanges of carbon, moisture, and energies have yet to be identified but prove essential to improve climate predictions further using bulk transfer models such as the Tropical Ocean and Global Atmosphere Coupled Ocean-Atmosphere Response Experiment (or TOGA-COARE) algorithm [2]. It is imperative to understand the fundamentals of the natural system of the tropical coastal ocean and how the transformation takes place over the time. Hence, latent and sensible heat fluxes, microclimate variables, and surface water temperature data (Fig. 1), collected over the periods of two years using eddy covariance instruments mounted on a platform at a tropical coastal ocean station is to gain the needful knowledge of the energy exchanges in the tropical climatic environment to further improve prediction algorithms. The parameters being measured are; (1) latent heat (LE); (2) sensible heat (H); (3) global radiation (RG); (4) net radiation (Rn); (5) wind speed (WS); (6) wind direction (WD); (7) atmospheric temperature (T), (8) relative humidity (RH), and (9) underwater temperature (Ts), Fig. 1 [3].

Therefore, the data has helped to determine the Monsoonal, and diurnal patterns of latent (LE), i.e., Fig. 1a, and sensible (H) heat (Fig. 1b), whereby the relationship between microclimate variables and the energy fluxes could be established.

## 2 Experimental design, materials and methods

Air-sea flux exchanges drive the climate and the global carbon-moisture cycle. Climate models forecast an increase in sea-surface temperature because of global warming, that subsequently intensifies the oceans׳ capacity to deliver heat to the atmosphere in the form of fluxes [3]. Ref. [4], suggested that the instrument and location responsible for these uptakes of energies are critical in altering global water budget and forecasting climate change realizable through direct measurement of the energy fluxes at the Atmosphere-Ocean interface.

Energy flux exchange in the tropical ocean requires further study as the magnitude of these fluxes have generally been estimated and seldom directly measured, except for a handful of studies for instance [5], [6], [7]. Some researchers hypothesized that an increase in evaporation is likely in tropical oceans due to high but relatively constant water surface temperatures. Ref. [8] reported persistent unstable atmospheric conditions above the tropical Lake Tanganyika, Africa, which could promote turbulent energy exchange between the air and water surfaces, that results in 13–18% energy loss through latent heat (evaporation) and sensible heat energy compared to neutral atmospheric conditions.

**Fig. 2:**
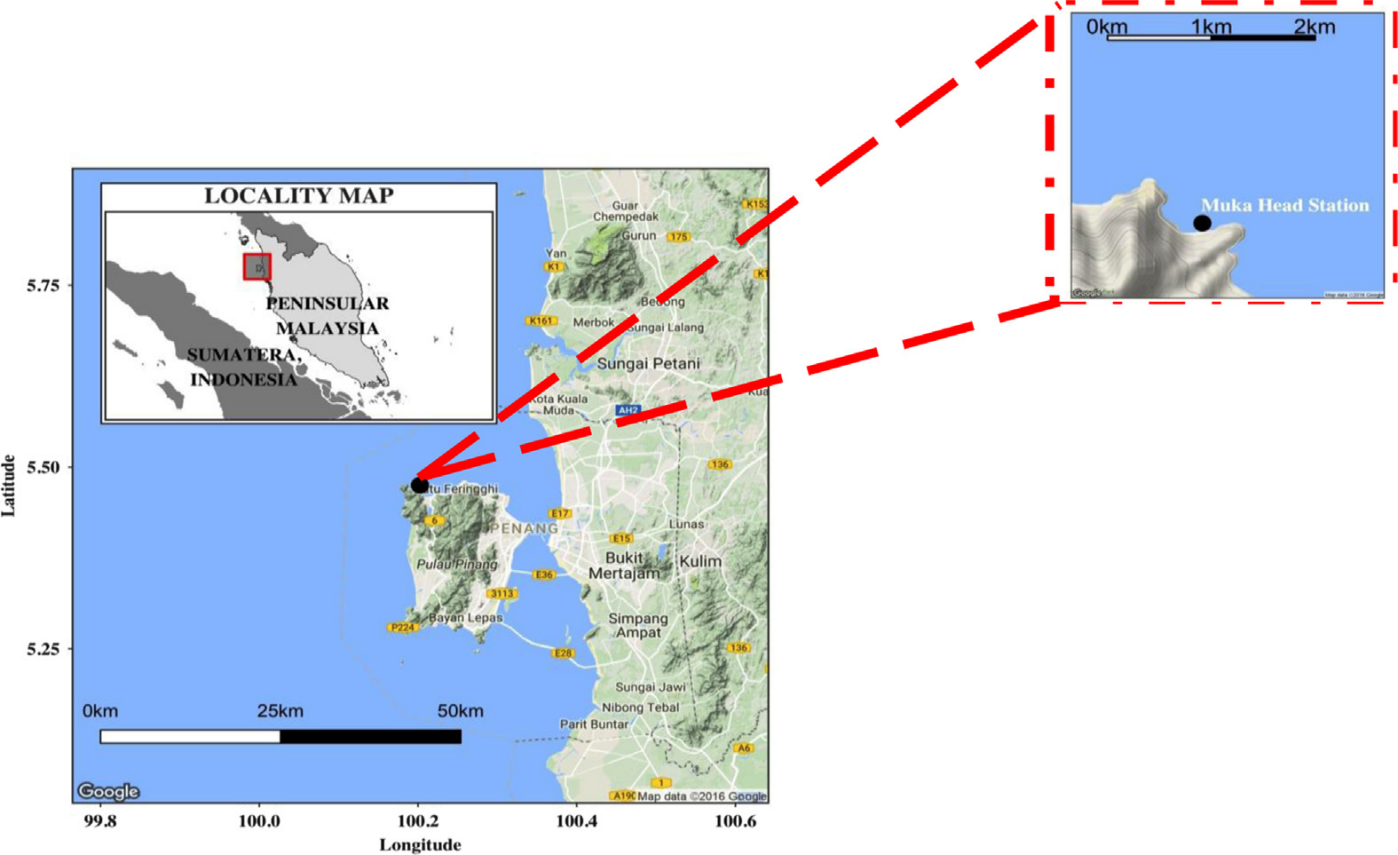
Data location map of the study site at (5°28′6′′N, 100°12′1′′E), denoted as the dark blue filled circle showing as the zoomed-in view at the top right panel.

Planet-scale studies of air-sea interactions ubiquitously depend on indirect flux quantification methods such as the bulk flux algorithm [9] and the Tropical Ocean and Global Atmosphere Coupled Ocean-Atmosphere Response Experiment (TOGA-COARE) algorithm [2]. These prediction methods are prone to energy budget imbalances and would result in flawed climate predictions using climate models that use these fluxes as their input. Incorrect energy budget approximations would also affect evaporation and precipitation estimates, which is directly related to ocean density and ocean circulation. Even so, most in-situ observations, buoys with weather sensors installed located in the ocean, employ the bulk flux method, possibly due logistics and monetary constraints. Research on accurate quantification of fluxes to relate the direct measurements of fluxes to indirect measurements [10], is ongoing. The best method to quantify fluxes is the direct measurement of these fluxes using the eddy covariance method (Fig. 2).
